# Assessing the quality of patient handovers between ambulance services and emergency department – development and validation of the emergency department human factors in handover tool

**DOI:** 10.1186/s12873-022-00567-y

**Published:** 2022-01-19

**Authors:** Marina Golling, Wilhelm Behringer, Daniel Schwarzkopf

**Affiliations:** 1 Centre of Emergency Medicine, Jena University Hospital, Am Klinikum 1, 07747 Jena, Germany; 2 Department of Emergency Medicine, Medical University of Vienna, Spitalgasse 23, 1090 Vienna, Austria; 3 Department of Anaesthesiology and Intensive Care Medicine, Jena University Hospital, Am Klinikum 1, 07747 Jena, Germany

**Keywords:** Emergency department, Handover, Human factors, Teamwork, Non-technical skills, Communication

## Abstract

**Background:**

Patient handover between prehospital care and the emergency department plays a key role in patient safety. Therefore, we aimed to create a validated tool for measuring quality of communication and interprofessional relations during handover in this specific setting.

**Methods:**

Based on a theoretical framework a comprehensive item pool on information transfer and human factors in emergency department handovers was created and refined in a modified Delphi survey involving clinical experts. Based on a pre-test, items were again revised. The resulting Emergency Department Human Factors in Handover tool (ED-HFH) was validated in a field test at the emergency department of a German university hospital from July to December 2017. The ED-HFH was completed by emergency department and ambulance service staff participating in handovers and by an external observer. Description of item characteristics, exploratory factor analysis, analyses on internal consistency and interrater reliability by intraclass-correlation. Construct validity was analysed by correlation with an overall rating on quality of the handover.

**Results:**

The draft of the ED-HFH contained 24 items, 90 of 102 eligible staff members participated in the field test completing 133 questionnaires on 38 observed handovers. Four items were deleted after analysis of item characteristics. Factor analysis supported a single factor explaining 39% of variance in the items. Therefore, a sum-score was calculated with a possible range between 14 and 70. The median value of the sum-score in the sample was 61.5, Cronbach’s *α* was 0.83, intraclass-correlation was 0.52, the correlation with the overall rating of hand-over quality was *ρ* = 0.83 (*p* ≤ 0.001).

**Conclusions:**

The ED-HFH showed its feasibility, reliability and validity as a measure of quality of information transfer and human factors in handovers between ambulance services  and the emergency department. It promises to be a useful tool for quality assurance and staff training.

**Supplementary Information:**

The online version contains supplementary material available at 10.1186/s12873-022-00567-y.

## Background

Patient handover between the personnel of the ambulance service (AS) and the emergency department (ED) plays a key role in patient safety [1–3]. The successful transfer of information during the handover between the AS and the ED is a critical point, due to a possible loss of information when the AS leaves the ED [4–7]. Therefore, several studies have been conducted to capture the transfer of information and to increase the congruence of transferred data between the AS and the ED [4, 7–10]. Intervention studies have shown that training in the use of mnemonics techniques and the standardisation of data might lead to more reliable information transfer [11]. Nevertheless, the effects of these interventions have mostly fallen short of expectations and research in this area provides contradictory results [12–14]. Focusing only on information transfer does not take into account the complexity of a handover, since information transfer cannot be separated from the human factors (HF) defined as psychological, cognitive and social characteristics of people influencing their interactions with the environment [5, 15–17]. Several studies on care transitions and on patient handovers in the prehospital setting showed that the quality, efficiency, and safety of handovers are significantly influenced by the social interactions and the quality of interpersonal relationships between the professional groups involved [16–18].

Validated observation and survey tools have been developed to assess HF in the context of handovers for several interfaces in the hospital [17, 19–21], but these do not sufficiently take into account the specifics of patient handovers from the AS to the ED. Therefore, the aim of this study was to develop and validate a questionnaire focusing on the HF of handovers, which is tailor-made for the emergency department: The Emergency Department – Human Factors in Handover tool (ED-HFH).

## Methods

This study reports the development and validation of a new standardized tool to assess HF during handovers between the AS and the ED. This tool was intended for use as a self-assessment questionnaire by participants of handovers as well as a tool for usage by an external observer. This validation study primarily focused on its usage as a questionnaire. The study was approved by the ethics committee of the Jena University Hospital (reference number 5093–02/17); all methods were carried out in accordance with relevant guidelines and regulations. Staff was asked for informed written consent for participation in observation and surveying in the context of handovers. Participation was voluntary.

### Development of the questionnaire

The development of the questionnaire was based on a theoretical framework including relevant constructs in the context of patient handovers derived from the literature and discussions with four experts from the AS and the ED: a) Active listening, i.e. bidirectional communication, b) mutual appreciation and respect as perceived by the participants [4, 22], c) professional relationships, defined by “thoughtfulness, reliability and clinical accuracy” [23, 24], d) teamwork including cooperation, coordination and the absence of conflicts, e) situational awareness as a shared mental model of the patient’s condition and its implications [21] and f) information transfer [25].

Items for measuring these constructs were mostly taken from the handover performance tool of Pezzolesi et al. [21] and adapted to the context of ED handovers. If original items were in English, they were translated to German and the translation was checked by three sworn translators for the English language. Where no items to measure intended constructs could be found, new items were designed by the first author. In addition, five items adapted from Pezzolesi et al. were used to assess contextual influences on the handover [21]. Two representatives of each professional group involved in the patient handover were recruited from the personal network of the authors and included in the Delphi process: Two doctors working in the ED and also working in the air ambulance services, two nurses, and two paramedics. Experts were selected either because they had practical experience both in the ED and AS, or because they had received additional scientific training. This way of recruitment and selection of experts is common for a Delphi process [26]. This set of initial items went through an adapted Delphi process conducted in two passes including six long-serving and experienced staff members of ED and the AS. In the first run, the experts received the item pool and were asked to rate the proposed items on a five-point scale regarding relevance (5 = relevant, 1 = irrelevant) and to write down comments and alternative wording, if necessary. All items, for which an alternative wording was proposed or the mean value or the modal value of relevance were below three, were revised. In the second run, the revised items again were presented to the experts. As mean and modal values of each item were higher than four and no alternative formulations were suggested, the Delphi-process was considered to be completed [26]. By involving experts and staff at this early stage of development, face validity and feasibility of the tool, as a measure of relevance and plausibility of the selected items, were assured [27].

This resulted in the first draft of the questionnaire containing 24 items. A Likert scale (1 = totally disagree; 5 = totally agree) with the additional option “irrelevant “was used. The draft was then pre-tested on 14 handovers with 28 participants from the ED and the AS. After evaluation of the descriptive statistics, formulations that showed a ceiling or floor effect (percentage of participants choosing lowest or highest possible ratings) were sharpened. Table 1 presents the final set of items used in the field test. The revised and validated questionnaire can be found as Additional file 1.

**Table 1:** Constructs and items of the ED-HFH tool and their item characteristics in the field test

Construct	Item	Median [1st quartile; 3rd quartile]	Mean ± SD	Mode	Range	Floor, No. (%)	Ceiling, No. (%)	Not relevant, No. (%)
Teamwork	1. All relevant information was shared between the ED and Ambulance team	5 [4;5]	4.5 ± 0.8	5	2–5	0 (0)	87 (65.9)	1 (0.8)
Teamwork	3. Ambulance service and ED team jointly assured the handover was complete.	4 [4;5]	4.2 ± 1.0	5	1–5	2 (1.5)	64 (48.5)	1 (0.8)
Teamwork	4. A good and collegial contact was established actively at the beginning of the handover.	5 [4;5]	4.3 ± 0.9	5	1–5	1 (0.8)	68 (51.1)	0 (0)
Teamwork	7. In order to focus on the handover. Side activities were deliberately interrupted (e.g. moving the patient from one bed to another. Take off monitoring. undress).	4 [3;5]	4 ± 1.2	5	1–5	8 (6.5)	60 (48.4)	9 (6.8)
Teamwork	8.^e^ Tasks to be completed were assigned to the ED personal (e.g. completing monitoring. Venous catheter. Current medication).	4 [3;5]	3.9 ± 1.3	5	1–5	10 (8.7)	49 (42.6)	18 (13.5)
Teamwork	11.^i^ There were tensions within the teams during the handover.	1 [1;1]	1.3 ± 0.7	1	1–5	100 (76.3)	2 (1.5)	2 (1.5)
Information transfer	2. All needed written information was handed over (including patient chart. Medication protocol. Living will etcetera)	5 [4;5]	4.5 ± 0.8	5	2–5	0 (0)	84 (63.2)	0 (0)
Information transfer	10. The handover was a good opportunity for the person taking on responsibility for the patient to ask questions.	5 [4;5]	4.3 ± 0.9	5	2–5	0 (0)	71 (54.2)	2 (1.5)
Information transfer	12. The participants of the handover were asked to complete missing information and clarify outstanding issues.	4 [3;5]	3.6 ± 1.2	5	1–5	6 (5)	33 (27.3)	12 (9)
Information transfer	16.^e^ Concerns about risks to patient care concerning infection. Germs. danger to themselves or others were expressed.	4 [2;5]	3.5 ± 1.4	5	1–5	15 (13.5)	39 (35.1)	22 (16.5)
Information transfer	17.^e^ Actions to prevent adverse patient outcome were articulated.	4 [2;5]	3.3 ± 1.4	5	1–5	13 (13.1)	26 (26.3)	34 (25.6)
Situational awareness	5.^e^ Unfamiliar members of the teams introduced themselves to each other.	3 [2;4]	3.2 ± 1.4	5	1–5	14 (12.3)	28 (24.6)	19 (14.3)
Situational awareness	6. The new person responsible for the patient was clearly chosen.	4 [3;5]	3.9 ± 1.3	5	1–5	6 (4.7)	62 (48.1)	(4) 3
Situational awareness	15. The patient’s condition is evaluated from the emergency call until handover as: stable. Improving. deteriorating.	4 [4;5]	4.2 ± 1	5	1–5	2 (1.7)	60 (49.6)	12 (9)
Respectful interactions	9. The responsible persons listened very carefully.	5 [4;5]	4.5 ± 0.8	5	1–5	2 (1.5)	81 (60.9)	0 (0)
Respectful interactions	13.^ii^ The handover was objective at every moment.	5 [4;5]	4.6 ± 0.7	5	1–5	1 (0.8)	99 (74.4)	0 (0)
Respectful interactions	14. The patient perceiving the handover and listening to the participants was considered carefully.	4 [3;5]	4 ± 1.1	5	1–5	2 (1.7)	48 (39.7)	12 (9)
Respectful interactions	18. The handover was characterised by mutual respect.	5 [4;5]	4.5 ± 0.8	5	1–5	2 (1.5)	88 (66.2)	0 (0)
Working environment	19. There were personnel bottlenecks affecting the handover.	2 [1;3]	2 ± 1.3	1	1–5	62 (48.1)	11 (8.5)	4 (3)
Working environment	20. The ED Team was under time pressure	2 [1;3]	2.3 ± 1.3	1	1–5	46 (35.4)	10 (7.7)	3 (2.3)
Working environment	21. The ambulance service was under time pressure.	2 [1;3]	2 ± 1.1	1	1–5	58 (44.6)	7 (5.4)	3 (2.3)
Working environment	22. The handover was interrupted (by phone calls. Newly entering personal. Etc.)	1 [1;2]	1.7 ± 1.1	1	1–5	83 (63.8)	9 (4.6)	3 (2.3)
Working environment	23. The case handed over was very complex.	2 [1;3]	2.3 ± 1.2	2	1–5	36 (27.7)	8 (6.2)	3 (2.3)

ED-HFH: Human factors in handover tool. Number of questionnaires without external observer: 133. Missing values = 0%

Domain = thematic domain to which the questionnaire item belongs. Response options: 1 = strongly disagree. 2 = disagree. 3 = neutral. 4 = agree. 5 = strongly agree. Floor = proportion and number of participants choosing the lowest possible answer category. Ceiling = proportion and number of participants choosing the highest possible answer category. ^e^ = excluded. > 10% rated irrelevant. ^i^ = included despite floor effect. ^ii^ = included despite ceiling effect

### Field test

#### Setting and conduction of the field test

The ED of the Jena University Hospital treats approximately 30.000 patients per year (average of 82 patients per day), of which 33% are brought in by the AS. Depending on the severity of symptoms, the patients are assigned to one of the 13 ED monitor stretchers, one of the three resuscitation/trauma rooms, or one of the six examination rooms.

Data collection in the emergency department took place from July to December 2017. During each shift, handovers were observed consecutively and unannounced. After completion of the patient handover, the questionnaire was handed out to all participants. Each handover was additionally observed by the first author using the same questionnaire as an observational tool.

### Analysis

#### Descriptive analysis of items

A descriptive analysis of the questionnaire items was performed to describe their distribution. The frequency of non-responders and floor and ceiling effects were examined. Items showing more than 10% nonresponse or rating “irrelevant” and more than 70% endorsement of highest or lowest category as indicators of poor statistical discrimination were considered for removal [28].

#### Exploratory factor analysis

Exploratory factor analysis based on the polychoric correlation matrix was conducted to investigate the relationship between the items of the questionnaire and the proposed theoretical constructs. This determines factorial validity, which is an aspect of construct validity [27]. The factor analysis involved items on HF and information transfer, excluding items measuring contextual factors. Factor analysis was based on the answers by staff excluding the external observer and followed the steps recommended in the literature [29]. Squared multiple correlations and non-rotated principal component analysis was used to check for multicollinearity or singularity among items. The Kaiser–Meyer–Olkin criterion and the measure of sampling adequacy were used to check factorability. Different criteria were applied for the identification of the number of factors: The number of eigenvalues greater than 1 in non-rotated principle component analysis and primary axis factoring, examination of the scree plots of principle component analysis and primary axis factoring, and parallel analysis. A series of orthogonal and oblique rotated primary axis factoring analyses using differing number of factors was conducted to find an optimal theoretically sound solution with simple structure of factor loadings.

#### Calculation of scales

Scales were calculated as the sum of the respective items as identified by FA. Missing values on items were handled by calculating the scale value based on the non-missing items as long as a maximum of one third of items was missing.

#### Reliability and inter-rater reliability

Reliability of scales was calculated using Cronbach’s alpha as a measure of internal consistency [27]. Cronbach’s alpha above 0.7 is regarded as acceptable. Since the ED-HFH does not measure properties of the individual but a shared experience of a social interaction, also interrater reliability of the scales needed to be investigated as a measure of agreement [30]. Inter-rater reliability assesses the level of similarity between judgements on the same objects (the handovers) by different judges (the participants in the handovers). The intra-class correlation (ICC) is the most commonly used metric to calculate inter-rater agreement for continuous variables [30]. An adapted version for calculation of the intra-class correlation was used to adjust for the fact that not all handovers were assessed by exactly the same participants [31].

#### Validity analysis

Construct validity of a measure can be shown by investigating, if relations to other measures conform to theoretical expectations [27]. We expected that a positive judgement on HF in handovers with the ED-HFH would show a positive relation to the overall rating of the quality of the handover by staff measured on a scale from 1 -worst possible handover to 10- best possible handover. After aggregating the values of the ED-HFH sum-scores by taking the mean rating for each handover, the Spearman’s correlation between the ED-HFH sum-score and the overall rating was calculated. Analyses were conducted using R version 3.6.1. (R core team, 2019).

## Results

Of the 102 AS and ED team members approached, 100 agreed and 88% participated in the study. Table 2 presents the characteristics of participating staff. There were no significant differences in the age structure between the AS and the ED. The proportion of physicians at the ED was higher than in the AD (*p* = 0.002). Overall, the staff in the AS had more professional experience than the staff of the ED (*p* = 0.047). The proportion of women in the emergency department was higher than in the ambulance services (*p* = 0.025).

**Table 2:** Characteristics of staff participating in the field test

	Full sample *N* = 90	Ambulance services *N* = 52	Emergency department *N* = 38	*P*-value
Profession				0.002
No physicians	63 (70)	43 (61.4)	20 (52.6)	
Physicians	27 (30)	9 (31.0)	18 (47.3)	
Years in practice				0.047
0–2 years	17 (18.9)	5 (9.6)	12 (31.6)	
3–5 years	20 (22.2)	11 (21.2)	9 (23.7)	
6–10 years	21 (23.3)	14 (26.9)	7 (18.4)	
> 10 years	32 (35.6)	22 (42.3)	10 (26.3)	
Sex				0.025
Female	33 (36.7)	14 (26.9)	19 (50)	
Male	57 (63.3)	38 (73.1)	19 (50)	
Age				0.064
< 25	7 (7.8)	4 (7.7)	3 (7.9)	
25–35	49 (54.4)	26 (50)	23 (60.5)	
36–45	21 (23.3)	10 (19.2)	11 (28.9)	
46–55	12 (13.3)	11 (21.2)	1 (2.6)	
> 55	1 (1.1)	1 (1.9)	0 (0)	

Descriptive data are given as number (%); significance testing is by χ2 test or Fisher’s exact test, as appropriate

A total of 38 handovers were observed and the staff survey was conducted; of 135 questionnaires handed out, 133 (99%) were answered. Handovers were rated by a mean number of 3.5 participants (range: 2–5); staff members took part in an average of 1.5 handovers (range: 1–6). All handovers were also assessed by the first author as external observer.

### Item characteristics

Table 1 shows the descriptive statistics for the questionnaire items. One item each showed a floor (item 11, 76.3%) or a ceiling effect (item 13, 74.4%). Items 5, 8, 16 and 17 were rated as irrelevant by more than 10% of the participants. Due to the great theoretical importance of items 11 and 13, they were kept for further analyses. Items 5, 8, 16 and 17 were excluded.

### Exploratory factor analysis

After assessment of the item characteristics, 14 items were entered in the factor analysis. A Kaiser-Meyer-Olkin criterion of 0.79 and measures of sampling adequacy of items between 0.62 and 0.89 indicated acceptable to good factorability [29]; no indications of multicollinearity or singularity among items were found. While PCA showed three principal components with an eigenvalue above 1, both scree test and parallel analysis suggested a one-factor solution (Additional file 2). Therefore, a one-factor primary axis factoring was calculated. Table 3 shows factor loadings and communalities of items for this solution. The factor explained 39% of the total variance of the items.

**Table 3:** Results of factor analysis: One-factor solution

Domain	Item	Loading	h2
Teamwork	1. Information exchange	0.69	0.48
Teamwork	3. Making sure handover was complete	0.7	0.48
Teamwork	4. Establishing good collegial contact	0.66	0.43
Teamwork	7. Interruption of side activities	0.51	0.26
Teamwork	11. Tensions within teams	−0.54	0.3
Information transfer	2. Handing over written information	0.54	0.29
Information transfer	10. Checkback opportunity	0.72	0.52
Information transfer	12. Completing missing information	0.59	0.35
Respectful interactions	9. Careful listening	0.78	0.6
Respectful interactions	13. Objective Handover	0.68	0.46
Respectful interactions	14. The patient’s perception	0.47	0.22
Respectful interactions	18. Mutual respect	0.73	0.53
Situational awareness	6. New person responsible for the patient clearly chosen	0.49	0.24
Situational awareness	15. Evaluation of the patient’s condition	0.59	0.35

Factor analysis based on *n* = 95 cases with complete data. Factors were extracted by principal axis factoring. Overall variance in items explained by factors was 39%. h2: Communality for the item

### Reliability

A Cronbach’s *α* of 0.83 (95% CI: 0.79, 0.87) indicated a good internal consistency of the resulting ED-HFH sum-score. The intraclass-correlation was 0.52, which indicated a substantial interrater-reliability [30].

### Construct validity

**Fig. 1:**
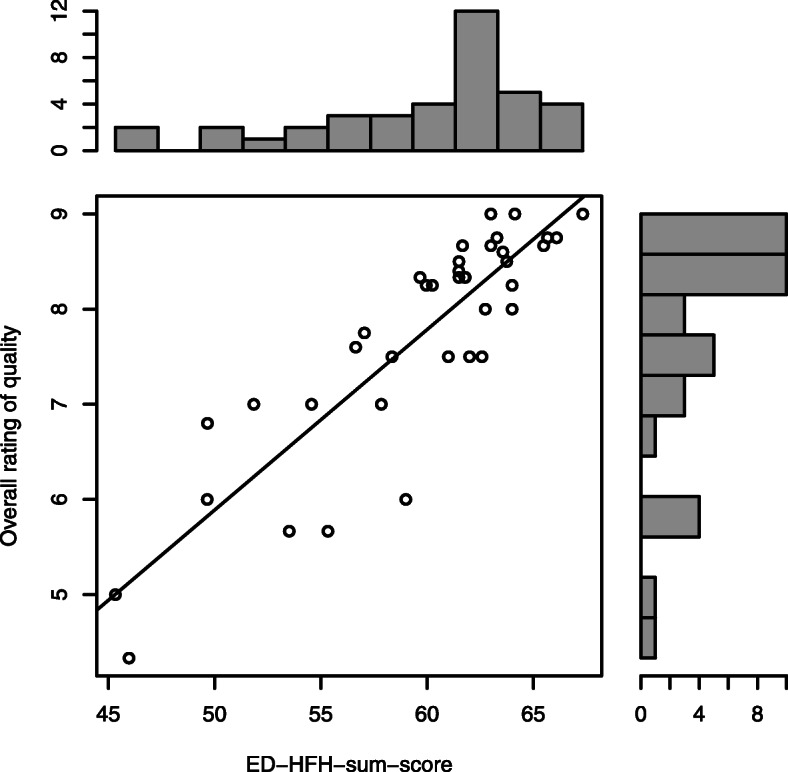
Relation between the ED-HFH sum-score with an overall rating of quality of handovers as rated by staff. Figure is based on 38 handovers and presents aggregated values calculated as the mean ratings of participating staff per handover

The handover aggregated HD-HFH sum-score showed a median of 61.5 [1st quartile: 57.25, 3rd quartile: 63.22] given a possible range from 14 to 70. This indicates highly positive ratings of HF and information transfer with little variation across handovers. The same was true for the HD-HFH sum-score rated by the external observer (58.5 [57, 63]) and the overall rating of the quality of the handover (8.25 [7.12, 8.57], with a possible range from 1 to 10). The Spearman’s correlation between sum-score and overall rating from staff was high (*ρ* = 0.83, 95%-CI: 0.7–0.91, *p* ≤ 0.001, Fig. 1).

## Discussion

This study presents the development and validation of a new tool for assessing HF in handovers between AS and the ED. The ED-HFH showed good feasibility both for self-assessment and as an observation tool. Face and content validity were assured by involvement of experts and staff from ED and AS in its development. In a field test, the ED-HFH showed good reliability and proofs of validity when used as a self-assessment tool by staff.

Most existing tools on patient handover including items on human factors come from the field of perioperative medicine [20, 32], shift handovers [23, 33], or are universally structured in order to be able to be used at different interfaces [17, 21]. Existing tools for the handover between AS and ED, put little emphasis on HF and concentrate on the quantity of data transferred [3, 9]. Therefore, the newly developed tool closes a gap for measuring quality in handovers between AS and the ED by integrating both items on information transfer and HF. In addition, some aspects that have repeatedly been claimed to be important for a successful patient handover and which have not been taken into account in existing tools, were integrated as new items, which are in particular: active listening [4], paying attention, [4, 22] and mutual respect including professional behaviour and objectivity [34].

The ratings on the items of the ED-HFH by participating staff as well as the observer were mostly positive. This might be representing a ceiling effect; i.e. the selected items might not be suited to assess existing variation between different handovers. However, only two items were considered for exclusion because of extreme ratings. Positive ratings might also be explained by an actual high quality of observed handovers, as was also indicated in the positive ratings of overall quality. Also other tools on measuring quality of handovers showed largely positive ratings [17].

Although the items of the ED-HFH were expected to represent four constructs, factor analysis showed only one common factor, which explained 39% of total variation in items. There are few studies on handover tools reporting a factor analysis for tool validation. The explained variation by the factors there ranged between 49% and 60% [17, 35]. The tool developed by Pezzolesi et al. has the greatest similarity to the ED-HFH. In her study, three correlated factors were identified accounting for 66% of variation [21]. The factor analysis in the study of Pezzolesi et al. was based mostly on ratings given by two trained observers. Trained observes might have a higher ability to distinct between different aspects of handover communication compared to staff giving a self-assessment without special training. Since only one external observer was available in this study (medical student without special training for observation of handovers) only the self-assessment data were used for this factor analysis. In addition, the Pezzolesi study focused on shift handovers between two physicians from the same department, while handovers between AS and ED involve several staff members from different professions and disciplines. This might increase the total variability in the data, thereby reducing the amount of variation explainable by the factor analysis. In sum, we believe our finding of a one-factor solution does not put the general construct validity of the tool in question, but reflects the judgemental processes in a self-assessment of participants in a handover between ED and AS.

The interrater-reliability of the ED-HFH was lower than Pezzolesi’s, where the ICC was between 0.75 and 0.88 [21]. Also this result can be explained by the comparison of data from trained observers. Likewise, other studies reporting higher ICCs used trained observers or tools with greater standardization [20, 36]. To our knowledge, this is the first validation study on a self-assessment tool on handover quality at this particular interface that actually examined interrater-reliability. Compared to other staff-questionnaires, which measure aspects of teamwork in healthcare [30], the interrater-reliability of the ED-HFH can be regarded as good.

### Limitations

The study was conducted in one study centre, limiting its generalizability. This limitation is shared with most comparable studies both regarding shift handovers [21, 35], handovers from the operating theatre [20, 36] or between AS and ED [3, 9, 37]. Therefore, future studies for validation of the ED-HFH should be multi-centric with larger sample sizes. This would also allow comparing different institutions regarding the quality of handovers. This study was only able to show the feasibility, but not the objectivity and validity of the ED-HFH for the usage by an external observer. Future validation studies should include at least two observers trained on standardized observation of handovers. Finally, the validity of the ED-HFH has been proven by a correlation with another self-rating of quality of the handover. Future studies should prove criterion validity, by assessing the relation between the ED-HFH and outcomes that are expected to be influenced by the quality of handovers, e.g. patient safety, morbidity and mortality.

## Conclusion

The ED-HFH promises to be a feasible tool for measuring and improving the quality of patient handover processes in the ED. This study has shown its feasibility, reliability, as well as content and construct validity. The ED-HFH also promises to be a short and useful tool for ongoing quality assurance of the handover in the ED [34]. It can also be part of a set of outcome measures to evaluate interventions to improve interprofessional cooperation [19, 20, 36]. Finally, it could be used as a tool for feedback and self-reflection in teaching interprofessional communication during handovers to students or medical staff [38].

## Supplementary Information

**Additional file 1.** (Questionnaire English).

**Additional file 2.** (Results of parallel analysis).

## Data Availability

All data requests should be submitted to Dr. Schwarzkopf (Daniel.schwarzkopf@med.uni-jena.de) for consideration. Access to the anonymised data might be granted following review by the data protection officer and the employee representation of the Jena University.

## Abbreviations

AS: Ambulance service
ED: Emergency department
ED-HFH: Emergency Department Human Factors in Handover tool
HF: Human factors
ED-HFH: Emergency Department Human Factors in Handover tool
ICC: Intra-class correlation
PCA: Principal Component Analysis
