# Supplementary material for: Assessing the quality of patient handovers between ambulance services and emergency department – development and validation of the emergency department human factors in handover tool
Source: BMC Emerg Med. 2022 Jan 19;22:10. doi: 10.1186/s12873-022-00567-y (PMC8772155; doi:10.1186/s12873-022-00567-y)
Supplement: Supplementary file 1 — Additional file 1. (Questionnaire English). [file 12873_2022_567_MOESM1_ESM.docx]

**Questionnaire on patient handover from ambulance services to emergency department**

| **Human factors in handover – Emergency department/ ambulance services** | 1 2 3 4 5 |  | Not appl./ irrelevant. |
| --- | --- | --- | --- |
| 1. All relevant information was shared between the ED and Ambulance team. 2. All needed written information was handed over (including patient chart, medication protocol, living will, etcetera). 3. Ambulance service and ED team jointly assured the handover was complete. 4. A good collegial contact was established actively at the beginning of the handover. 5. The new person responsible for the patient was clearly chosen. 6. In order to focus on the handover, side activities were deliberately interrupted (e.g. moving the patient from bed to another, take off monitoring, undress). 7. The responsible persons listened very carefully. 8. The handover was a good opportunity for the person taking on responsibility for the patient to ask questions. 9. There were tensions within the teams during the handover. 10. The participants of the handover were asked to complete missing information and clarify outstanding issues. 11. The handover was objective at every moment. 12. The patient´s perception of the handover considered carefully e.g. him or her listening to the participants. 13. The patient´s current condition is evaluated from the emergency call until handover as: stable, improving, deteriorating. 14. The handover was characterised by mutual respect. 15. There were staff shortages affecting the handover. 16. The ED team was under time pressure. 17. The ambulance service was under time pressure. 18. The handover was interrupted (by phone calls, newly entering personal etc.) 19. The case handed over was very complex. |  |  |  |
|  |  |  |  |
| 1. From 1-10, how would you rate the just completed handover?   1= worst, imaginable handover  10= best, imaginable handover |  |  |  |
| 1= strongly disagree, 3= neutral, 5 = strongly agree, Not appl = not applicable, irrelevant. | | | |
