# Supplementary material for: Assessing the quality of patient handovers between ambulance services and emergency department – development and validation of the emergency department human factors in handover tool
Source: BMC Emerg Med. 2022 Jan 19;22:10. doi: 10.1186/s12873-022-00567-y (PMC8772155; doi:10.1186/s12873-022-00567-y)
Supplement: Supplementary file 2 — Additional file 2. (Results of parallel analysis). [file 12873_2022_567_MOESM2_ESM.docx]

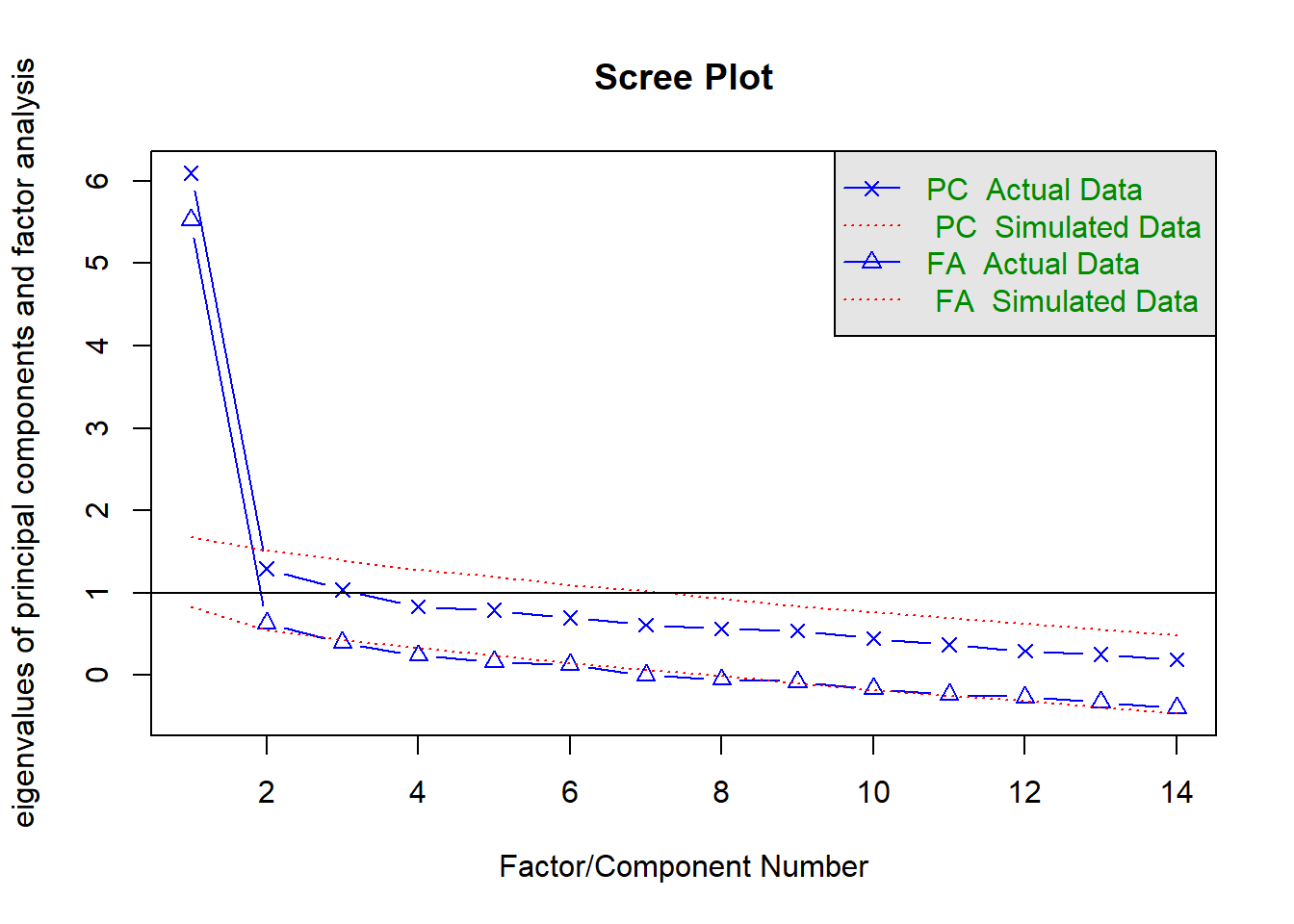


Supplemental Figure: Scree plot to represent the results of parallel analysis.
PC: principal component analysis, FA: factor analysis.
Three principal components showed eigenvalue > 1, one factor showed eigenvalue > 1, the scree test suggest one component or factor (number of factors/components left of the "elbow" of the graph), parallel analysis suggests one factor or one component (number of factors/components above the respective red-dotted line). Since most criteria suggested a 1-factor-solution a principal axis factoring analysis was conducted extracting one factor.
